# The Impact of COVID-19 on Trends of Violence-Related Offences in Australia

**DOI:** 10.1007/s44197-023-00131-2

**Published:** 2023-06-23

**Authors:** Peter Miller, Kira Button, Nicholas Taylor, Kerri Coomber, Ryan Baldwin, Travis Harries, Brittany Patafio, Tahnee Guala, Nathan Harris, Ashlee Curtis, Gery C. Karantzas, Petra K. Staiger, Dominique de Andrade

**Affiliations:** 1 School of Psychology, Deakin University, Geelong, Australia; 2 National Drug Research Institute, Curtin University, Melbourne, Australia; 3 Burnet Institute, Melbourne, Australia; 4 Griffith Criminology Institute, Griffith University, Brisbane, Australia

**Keywords:** COVID-19, Pandemic, Assault, Australia, Violence, Police

## Abstract

**Objective:**

To investigate the medium-term impacts of the COVID-19 pandemic on violence-related offences in Australia, and whether there was evidence of a ‘dual pandemic’ of family violence in addition to COVID-19.

**Methods:**

Autoregressive Integrated Moving Average time series were conducted to analyse publicly available violent crime statistics data from January 2017 to November 2021. Population rates of homicide, sexual, domestic and non-domestic assault were assessed across each Australian state and territory, with the effects of COVID-19 being modelled using the average monthly World Health Organization COVID-19 stringency rating for each jurisdiction.

**Findings:**

All jurisdictions in Australia showed increasing or stable domestic assault trends over the past decade, which were not significantly impacted by COVID-19, nor by the subsequent lockdowns. Non-domestic assaults demonstrated a significant, negative relationship with the stringency index for each jurisdiction, except Western Australia. There was no significant change in the rates of homicide or sexual assault across Australia in relation to COVID-19.

**Conclusion:**

Overall, there was no evidence of a ‘dual pandemic’ in Australia, and whilst domestic assaults continue to increase across the country, non-domestic assaults showed a notable but brief decline. However, these have returned to levels at least as high as pre-COVID-19 and some states show a continuing upward trend. The findings also suggest that alcohol availability may have played a role in continuing high violence numbers. Given the ongoing increasing and high levels of family violence in Australia, revised conceptual frameworks and interventions are indicated.

## The Impact of COVID-19 on Trends on Violence-Related Offences

The SARS-CoV-2 (COVID-19) pandemic and subsequent public health measures have placed unprecedented stress on billions of people worldwide [1, 2]. A recent meta-analysis determined that around a quarter of the general population experienced significant psychological stress due to the pandemic [3]. Psychosocial factors attributable to this stress include increased financial pressure, fear of infection, and the spread of misinformation [3–5]. When individuals experience substantial stress, their ability to make decisions and regulate emotions becomes impaired, sometimes this is associated with increased aggression [6]. There is evidence that stringent lockdowns and other restrictive measures also exacerbated alcohol use and social isolation in many countries [7–9]. The current study investigates the medium term impacts of the COVID-19 pandemic on four types of violence-related offences (domestic assault, non-domestic assault, sexual assault, and homicide) in Australia.

### Relevant State and Federal COVID-19-Related Restrictions/Measures

Table 1 outlines the various federal and state-level policy responses to the COVID-19 pandemic regarding restricted movement of people and economic support. New South Wales initiated the first State Government responses to the COVID-19 pandemic in mid-March 2020, cancelling all public gatherings of over 500 people. A nationwide ban on all non-essential mass gatherings shortly followed. On March 23, 2020, a nationwide 6-week lockdown was announced, including the temporary halting of all non-essential business and recreational activities. Social distancing measures were also introduced, initially limiting indoor social gatherings to ten people and, by the end of March, to two people. These measures remained in place across the nation until early May 2020. Between July 2020 and November 2021, all Australian states and territories were subject to varying lockdown measures in response to the pandemic. Notably, Victoria was subject to 16 continuous weeks of lockdown with stage 3 'Stay at Home' restrictions between April and October 2020 (the longest continuous lockdown worldwide), during which time only one person, per household, per day was allowed to leave home for one of four permitted reasons; 1) to collect essential food or supplies, 2) to receive medical care, 3) to attend essential work or education (if unable to do so from home), and 4) to exercise (for a limited time within a limited radius of your house). New South Wales also endured a similarly extended period of continuous lockdown restrictions (15 weeks) between June and October 2021. Outside of the Victorian and New South Wales extended lockdowns, most other states and territories employed shorter targeted lockdown measures in response to local outbreaks as they emerged, albeit with similar restrictions to those applied across Victorian and New South Wales. Over $223 billion in economic support was announced throughout March 2020 by federal and state governments combined.

**Table 1:** Government COVID-19-related interventions in Australia

Date	Jurisdiction	Measure
Restricted movements /lockdowns		
15 March 2020	New South Wales	Order under the Public Health Act 2010 (NSW) to immediately cancel all events with > 500 people
18 March 2020	New South Wales	Ban on non-essential indoor gatherings of ≥ 100 people (including staff)
16 March 2020	Federal	Australian Health Protection Principal Committee (AHPPC) banned non-essential mass gatherings
23 March 2020	Federal	Nationwide lockdown
30 June 2020	Victoria	Reinforced local lockdowns across ten Melbourne postcodes
4 July 2020	Victoria	Two additional Melbourne postcodes forced into lockdown
8 July 2020–27 October 2020	Victoria	Metropolitan Melbourne and Mitchell Shire re-enter lockdown
19–22 November 2020	South Australia	Lockdown
8–11 January 2021	Queensland	Lockdown
31 January–5 February 2021	Western Australia	Lockdown
12–17 February 2021	Victoria	Lockdown
29 March–1 April 2021	Queensland	Lockdown
24–27 April 2021	Western Australia	Lockdown
27 May–10 June 2021	Victoria	Lockdown
26 June–2 July 2021	Northern Territory	Lockdown
26 June–11 October 2021	New South Wales	Lockdown
12 August–14 October	Australian Capital Territory	Lockdown
5 August–21 October 2021	Victoria	Lockdown
29 June–3 July 2021	Western Australia	Lockdown
29 June–3 July 2021	Queensland	Lockdown
20–28 July 2021	South Australia	Lockdown
31 July–11 August 2021	Queensland	Lockdown
15–27 July 2021	Victoria	Lockdown
8–11 August 2021	Queensland	Lockdown
16–20 August 2021	Northern Territory	Lockdown
15–18 October 2021	Tasmania	Lockdown
5–9 November 2021	Northern Territory	Lockdown
Economic responses		
11 March 2020	South Australia	$350 million economic stimulus package
12 March 2020	Federal	$17.6 billion stimulus package
16 March 2020	Western Australia	$607 million economic response package for households and small businesses
17 March 2020	New South Wales	$2.3 billion health boost and economic stimulus package; including $700 million extra health funding $1.6 billion in tax cuts
17 March 2020	Tasmania	$420 million support package
18 March 2020	Northern Territory	$65 million jobs rescue and recovery plan
20 March 2020	Australian Capital Territory	$137 million economic survival package
21 March 2020	Victoria	$1.7 billion economic survival and jobs package
22 March 2020	Federal	$66 billion second stimulus package
24 March 2020	Queensland	$4 billion health, jobs, households, and businesses support package
31 March 2020	Federal	$130 billion “Jobkeeper” wage subsidy programme (later revised to $70 billion)

Source: Storen et al. [10]

### Impacts of COVID-19 Internationally

Internationally, one widely publicised repercussion of the COVID-19 pandemic is the potential of an increase in reports of family and domestic violence [11]; a ‘dual pandemic’. Several factors associated with family and domestic violence perpetration, such as male unemployment, alcohol use, and financial insecurity/stress, were intensified by the pandemic conditions [12]. Initial studies indicate that the prevalence of family and domestic violence increased in Europe, Asia, and America since the COVID-19 pandemic [11, 13–18]. In the Chinese Province of Hubai, domestic violence-related calls to the police increased approximately fourfold during lockdown [19]. In other countries, whilst formally recorded domestic violence-related offences and police involvement decreased, calls to domestic violence hotlines increased substantially [14, 20]. Commonly, a spike in family and domestic violence cases was observed immediately after a lockdown was implemented, before returning to relatively normal levels [18, 19, 21, 22]. One study from Japan found sexual assault victimisation significantly decreased during lockdowns, which may reflect the reduction of social interaction in public spaces such as public transport or bars [23].

Regarding non-domestic physical violence offences, preliminary studies indicate that rates declined as a result of the pandemic conditions (e.g. lockdowns) [24, 25]. Specifically, one large-scale study investigated the immediate effects of stay-at-home policies on crime across 23 countries in The Middle East, America, Europe, and Asia [24], reporting a 35% reduction in daily non-domestic assaults and a marginal reduction in homicides (only 3 cities showed significant reductions). These outcomes were relatively consistent across existing studies, although the magnitude of the decline varied [23, 25–27]. Further, more stringent lockdown measures were associated with greater reductions in most crime categories [24]. Although, research from the United States found that homicide rates during the pandemic remained at pre-pandemic levels [28, 29]. The reduction in non-domestic physical violence-related crime during the lockdowns was likely due to decreased mobility and changes in routine activity caused by lockdown measures, which may have increased guardianship and reduced offending opportunities [23, 24]. However, these same factors likely influenced the spike in domestic violence cases [19, 24].

Whilst there are a number of international studies examining the impact of the COVID-19 pandemic conditions on assault, homicide and sexual offences, there has been little Australian research published on this topic beyond the early months of the pandemic [9, 25, 30]. Payne and colleagues found that serious assault and sexual assault in Queensland declined significantly by the end of April 2020, although domestic violence breaches remained unchanged [25]. However, due to the differences in restriction measures implemented by jurisdiction, and the substantial demographic differences across the various states, this pattern of findings may not be generalisable to other Australian states and territories. Whilst there were a number of reports published on domestic violence specifically, none of these were peer reviewed and most reviewed have subsequently been removed from the internet. A qualitative and quantitative survey of 166 practitioners from Victoria, Australia [31] reported that the pandemic led to an increase in the frequency and severity of domestic violence alongside an increase in the complexity of women’s needs. They also reported that for many people experiencing violence during this period, there was a reduction in the ability to seek help and numerous challenges to providing supports for families at risk.

Therefore, the aim of the current study was to examine the medium-term impacts of the COVID-19 pandemic on violence outcomes for most Australian jurisdictions.

## Method

### Data

The current study utilises publicly available crime statistic data from each Australian jurisdiction [32–38]. The offence rates for three categories of violent crime (sexual offences, assault [domestic and non-domestic] and homicide) were obtained. The assault offence category was divided into domestic violence and non-domestic violence related offences in all jurisdictions, except for South Australia (SA), Queensland and Tasmania, where this differentiation was not available. The current study utilised offence rate per 10,000 population. Population rates were retrieved from the Australian Bureau of Statistics [39] and monthly offence rates between January 2017 and November 2021 were examined. The timeframe of the population rates retrieved (2017–2021) was based on the data that were publicly available from all jurisdiction. However, only yearly violent-offending statistics were available for Tasmania. It must be noted that data from different jurisdictions should not be compared directly as each has different protocols regarding what information is recorded, how that information is classified and how different offences are classified.

The monthly average stringency index (ranging from 0 to 100) rating for each jurisdiction from the Oxford COVID-19 government response tracker dataset [40] was included as the intervention variable in all models to account for the impact of the various COVID-19 restrictions introduced during the period examined. This dataset tracks COVID-19 policy responses in more than 180 countries and a higher score indicates more stringent restrictions.

As the current study is using publicly available, anonymous data, an ethics exemption was granted by the Deakin University Human Research Ethics Committee (HREC 2022-139).

#### Analysis Plan

Autoregressive Integrated Moving Average (ARIMA) time series analysis was used to estimate the influence of the stringency of COVID-19 policies on monthly offence rates. ARIMA was the most appropriate analysis to test the research hypotheses, given the dataset is time based and this approach can detect acute changes in trends [41]. The standard modelling approach for ARIMA analysis was used [41]. Where data exhibited clear positive or negative trends, first order differencing was used to transform the data into a stationary series. After differencing the data series, seasonality was assessed by examining the autocorrelation and partial autocorrelation plots; seasonal models were fitted where periodic trends were observed (e.g. a spike every 6 or 12 months; [42]). The auto-regressive and moving average values were determined by examining the autocorrelation plots and partial autocorrelation plots, respectively, and using reference plots to assign these terms to the ARIMA models [43]. No transfer function (commonly referred to as lag) were used in any of the models, as the impact of COVID-19 policies was expected to be immediate. All analyses were conducted using Stata version 17.0.

## Results

Data below are presented primarily by offence type (domestic assault, non-domestic assault, sexual offences, and homicide) where possible. However, where this is not possible, the data have been presented in the most efficient format.

### Domestic Assault

**Fig. 1:**
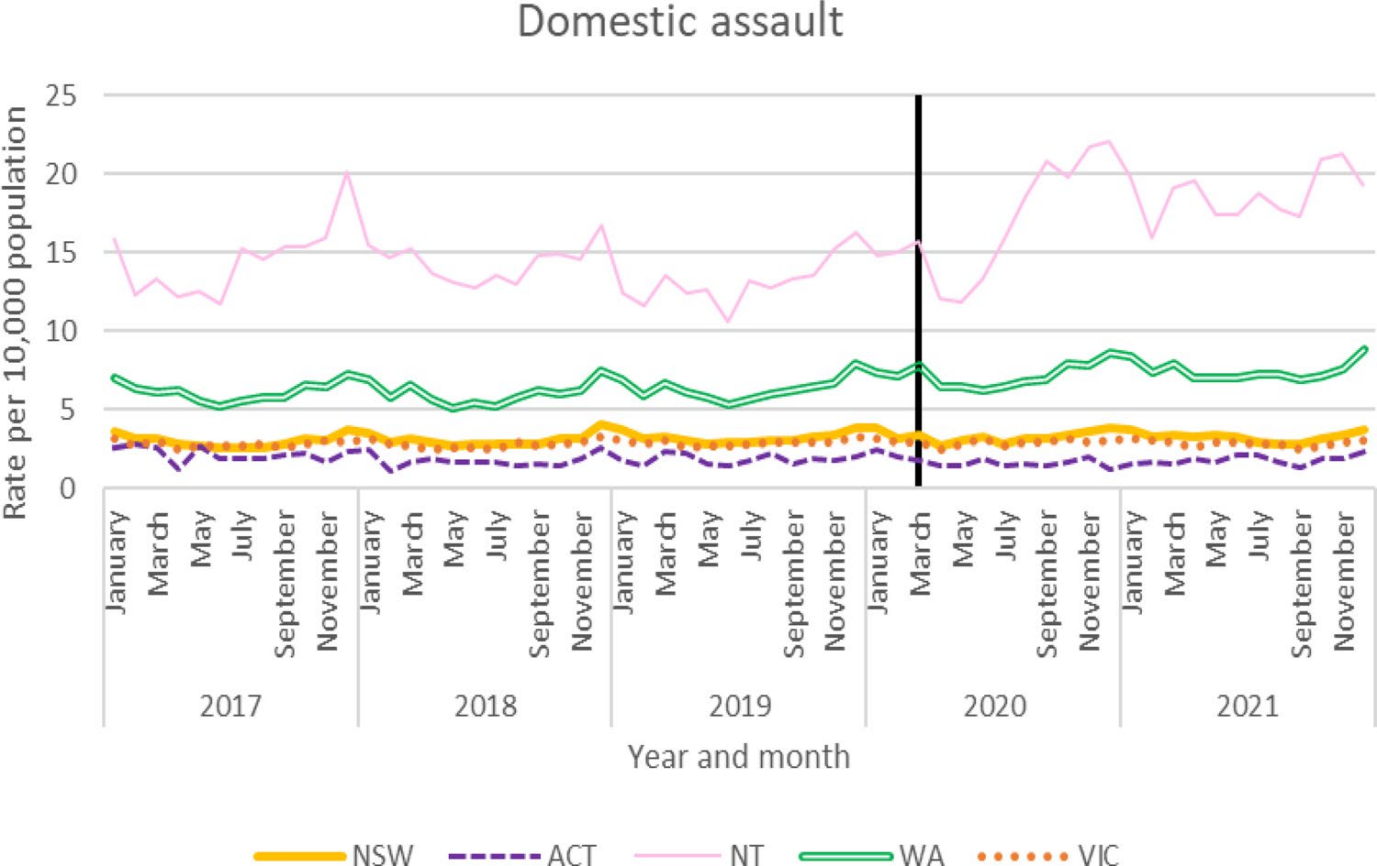
Domestic assault rates by jurisdiction

Monthly domestic assault rates were obtained for New South Wales (NSW), Australian Capital Territory (ACT), Northern Territory (NT), Western Australia (WA) and Victoria. As shown in Fig. 1, notable seasonal variation could be observed in WA, the ACT and NSW, wherein cases tended to peak towards the ends of the year and dip in April 2020, the month after the start of the first COVID-19 lockdown, and the first month that all of Australia was in lockdown. In the NT, there was a unique situation where many remote communities were put into ‘bio lockdown’ (mandated restrictions on the movement of people between communities/regions) and people were returned to community between March and May with little access to alcohol (most communities do not permit alcohol), whilst receiving substantially greater financial income via government economic stimulus schemes than normal. When lockdown ended, many individuals were seen to move to centres where alcohol was available and engaged in extended drinking sessions with substantial roll-on effects on violence and injury.

#### Time Series Analyses

ARIMA time series analyses were used to determine the impact of COVID-19 on Australian states and territory’s rates of domestic assault (Table 2). No jurisdictions showed significant changes in domestic assault across the period examined.

**Table 2:** ARIMA models for domestic violence police-recorded assaults

State	Model specification	*q*	*p*	Coefficient	*p*	Lower CI	Upper CI
Australian Capital Territory	(0,0,1) (0,0,1,4)	19.59	0.879	− 0.01	0.088	− 0.01	0.001
New South Wales	(1,0,0) (1,0,0,12)	12.67	0.994	− 0.002	0.06	− 0.01	0.0001
Northern Territory	(1,1,1)	34.36	0.156	− 0.06	0.089	− 0.12	0.01
Western Australia	(1,1,1) (1,1,1,12)	17.45	0.683	0.001	0.915	− 0.02	0.02
Victoria	(1,0,1) (1,0,1,12)	23.95	0.684	0.001	0.565	− 0.001	0.002

### Non-domestic Assaults

**Fig. 2:**
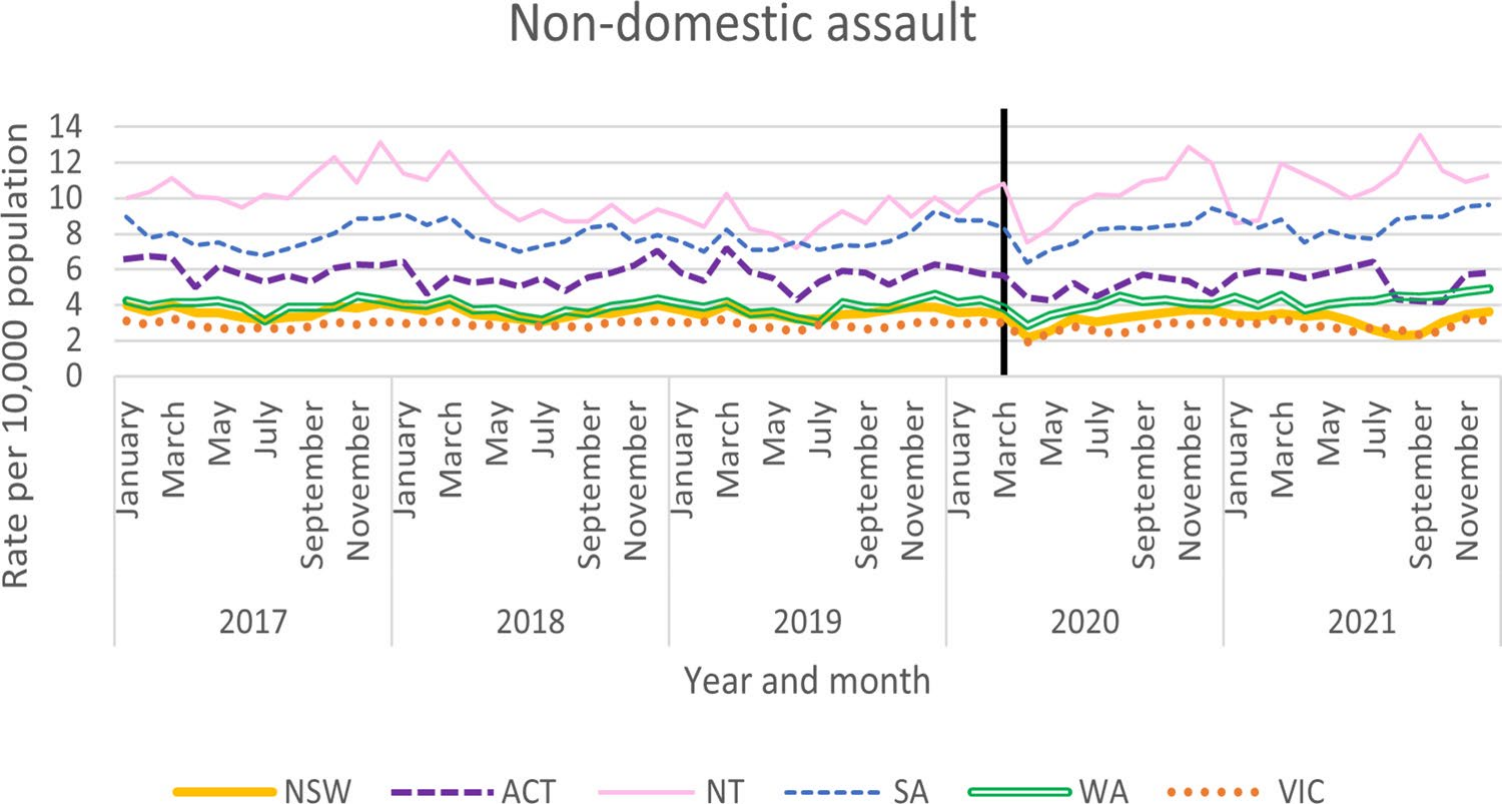
Non-domestic assaults by jurisdiction

Monthly non-domestic assault rates were obtained for NSW, ACT, NT, and WA. Monthly total assault rates (inclusive of domestic and non-domestic assaults) were also obtained from SA and included in Fig. 2. Notable seasonal variation was observed in NSW, ACT, SA and WA, wherein cases tended to peak towards the ends of the year and dip towards the middle of each year (see Fig. 2). This seasonal dip appears to have coincided with April 2020, the month after the start of the first COVID-19 lockdown, and first month that Australia was entirely in lockdown. However, it should be noted this dip appears to be uncharacteristically deep when compared to previous years.

### Combined Assault Data for Specific States

Not all data obtained from states were directly comparable. Monthly data could not be obtained from Tasmania, Victoria and SA. Some states, like Queensland only provide data for domestic and non-domestic assaults combined.

#### Queensland

**Fig. 3:**
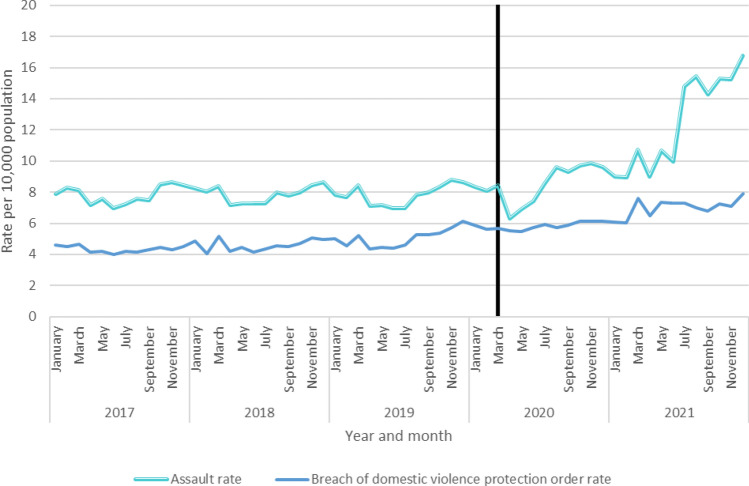
Recorded assaults and breach of domestic violence protection orders in Queensland

Due to a change in reporting practises for assault in July 2021, a large spike appears and is maintained in the assault trends (Fig. 3). Therefore, breach of domestic violence protection orders was included to provide an indication as to domestic violence trends within Queensland. It should be noted that the April 2020 dip in assault rates seen in other jurisdictions is also seen in Queensland.

#### Tasmania

**Fig. 4:**
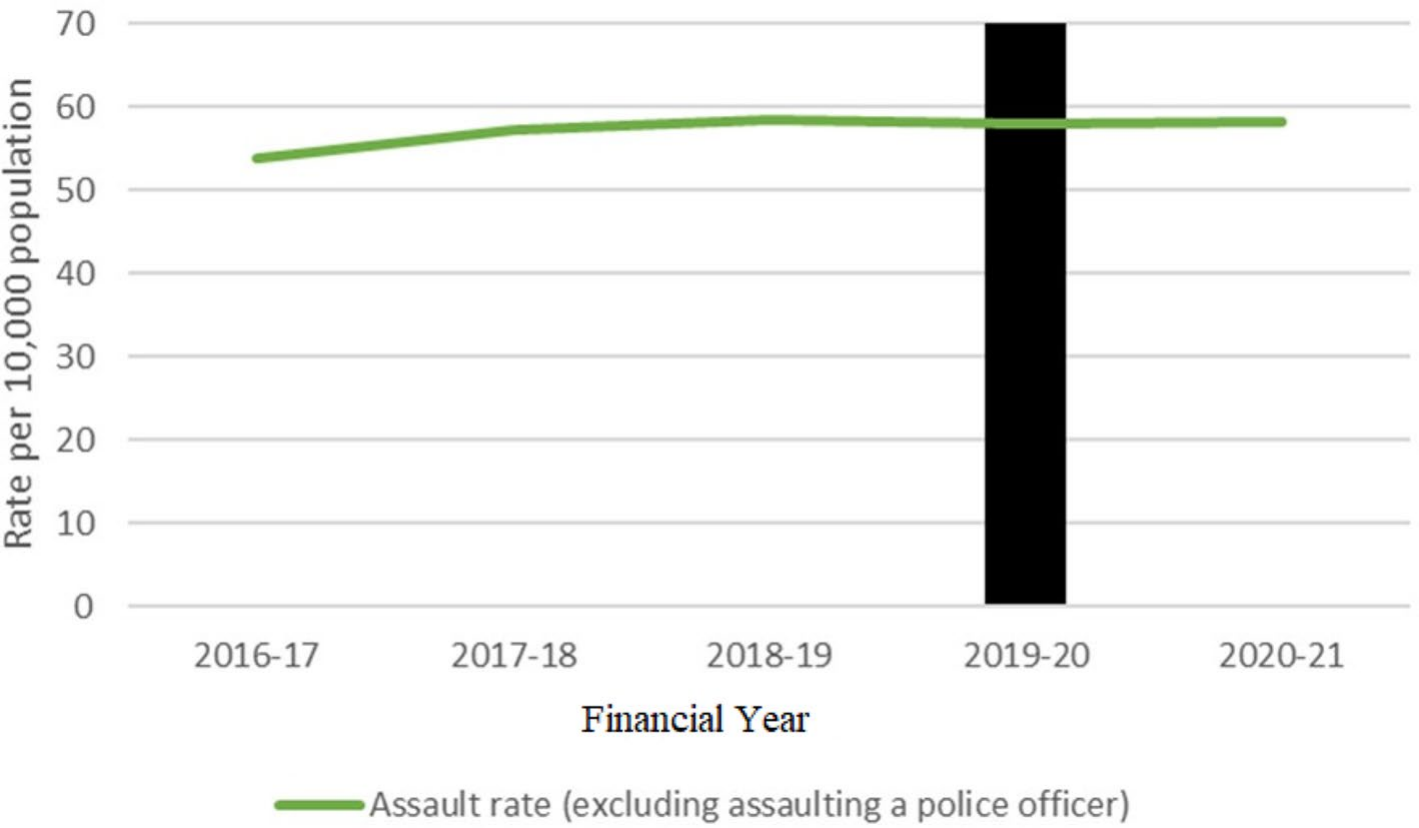
Recorded assaults in Tasmania

Financial year (1 July–30 June) assault rates were obtained from Tasmania. Rates remained stable across the period examined (Fig. 4).

#### South Australia

**Fig. 5:**
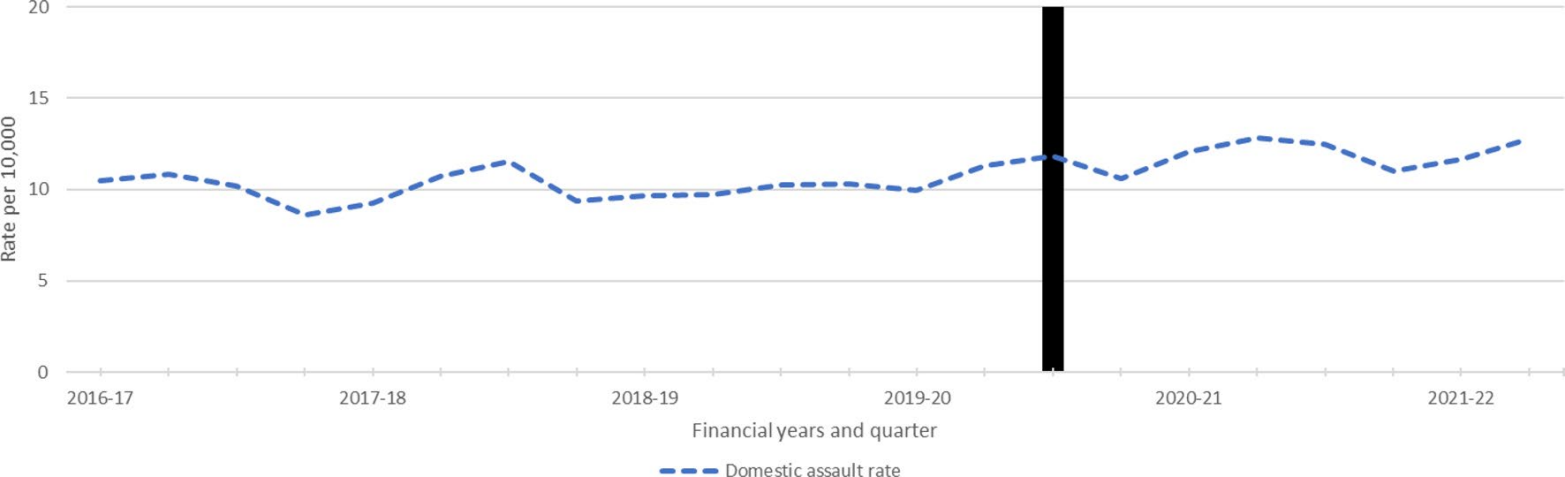
Recorded domestic assaults in South Australia

Quarterly domestic assault rates were obtained from SA, rates remained stable, although fluctuating, across the period examined (Fig. 5).

#### Time Series Analyses

ARIMA time series analyses were used to determine the impact of COVID-19 on Australian states and territory’s rates of non-domestic assault (Table 3). All jurisdictions, except WA, saw a significant decline in non-domestic assaults during the period examined. Stricter COVID-19 restrictions were significantly associated with lower assault rates in NSW, SA, NT, ACT and Victoria.

**Table 3:** ARIMA models for non-domestic assault

State	Model specification	*q*	*p*	Coefficient	*p*	Lower CI	Upper CI
Australian Capital Territory	**(0,0,1) (0,0,1,4)**	**24.89**	**0.63**	**− 0.01**	**0.001**	**− 0.02**	**− 0.01**
New South Wales	**(0,1,1) (0,1,1,12)**	**19.25**	**0.56**	**− 0.02**	** < 0.001**	**− 0.03**	**− 0.02**
Northern Territory	**(1,0,1)**	**35.03**	**0.16**	**− 0.03**	**0.041**	**− 0.05**	**− 0.001**
South Australia^a^	**(1,0,0) (1,0,0,12)**	**35.68**	**0.15**	**− 0.02**	**0.007**	**− 0.04**	**− 0.01**
Western Australia	(1,0,1) (1,0,1,12)	26.79	0.53	0.0001	0.986	**− **0.01	0.01
Victoria	**(0,0,1) (0,0,1,12)**	**33.28**	**0.22**	**− 0.003**	**0.018**	**− 0.01**	**− 0.001**

Bold text indicates significant results

^a^South Australia includes domestic assaults

### Sexual Offences

**Fig. 6:**
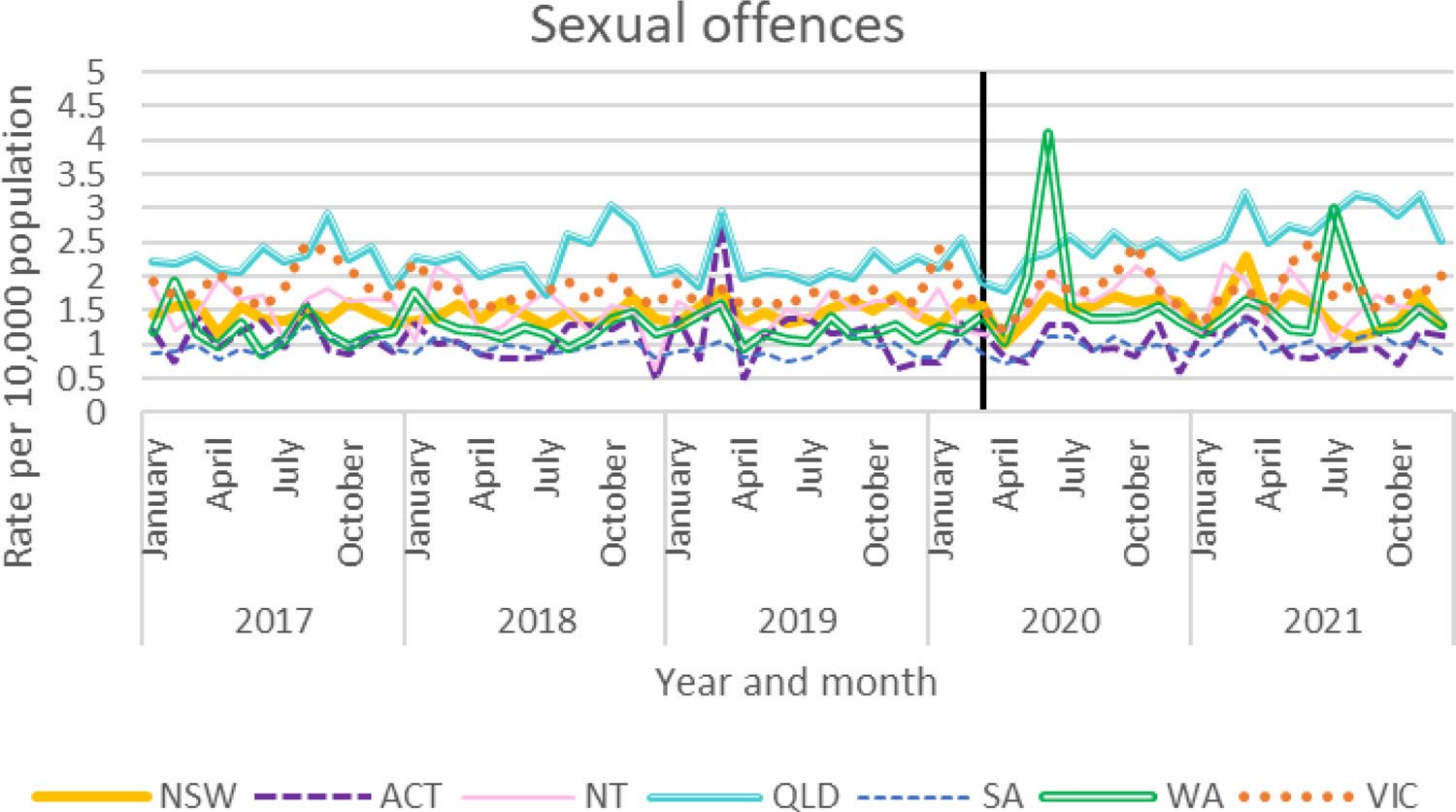
Recorded sexual offences by jurisdiction

**Fig. 7:**
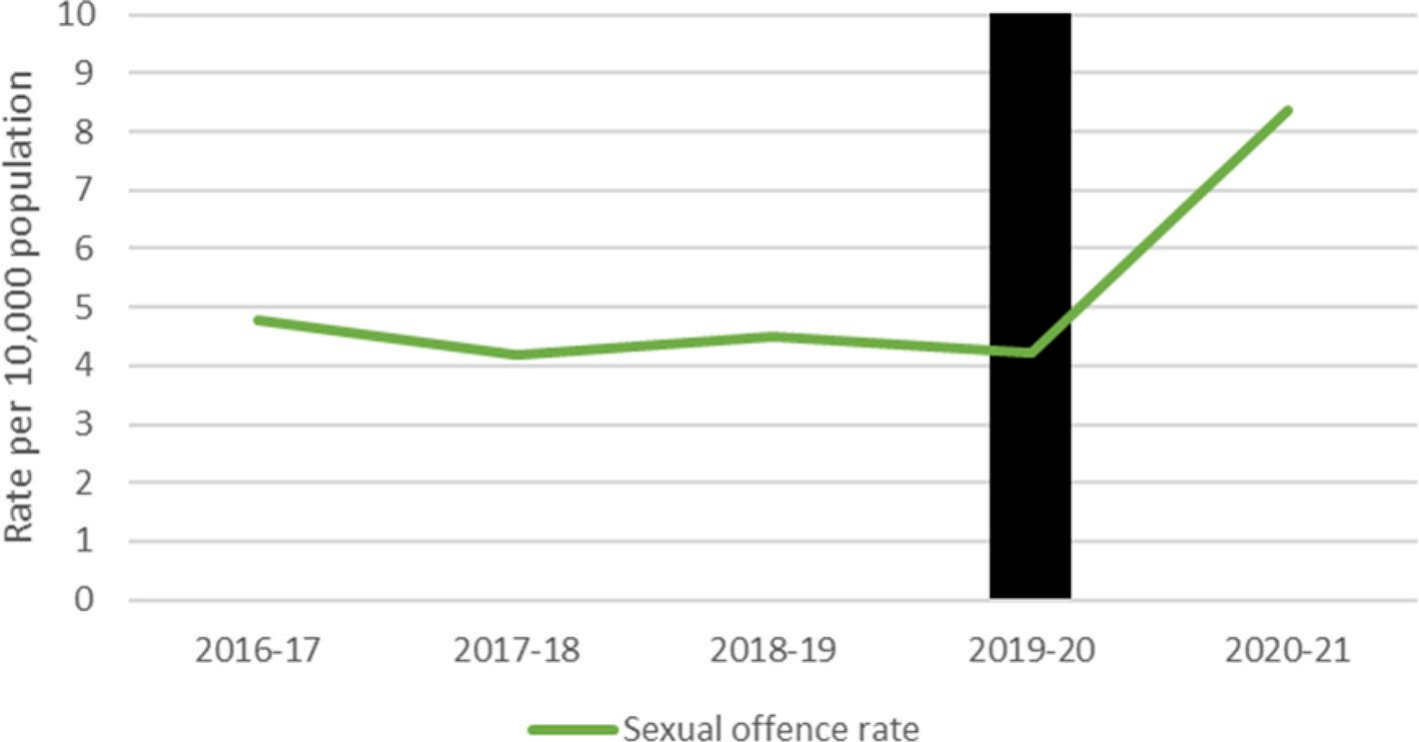
Recorded sexual offences in Tasmania by financial year

The results for sexual offences below report on monthly offence rates for all states and territories (Fig. 6), with the exception of Tasmania (which reports yearly; see Fig. 7). As shown in Fig. 6, trends remained stable across the period examined, although notably there were two large spikes in sexual offences in WA, both of which occurred when there were no restrictions in place.

#### Tasmania

Financial yearly sexual offence rates were obtained from Tasmania, which saw a large increase in the financial year following the first COVID-19 lockdown.

#### Time Series Analyses

ARIMA time series analyses were used to determine the impact of COVID-19 on Australian jurisdictions’ rates of sexual offences (Table 4). Sexual offence rates were stable across all jurisdictions during the time period analysed. A significant increase in sexual offences was seen in WA (results bolded); however, it should be noted that two anomalous spikes in sexual offences were recorded during the period when no restrictions were in place.

**Table 4:** ARIMA models for sexual offences

State	Model specification	*q*	*p*	Coefficient	*p*	Lower CI	Upper CI
Australian Capital Territory	(0,0,0)	26.79	0.53	− 0.01	0.078	− 0.01	0.001
New South Wales	(1,0,0)	40.32	0.06	− 0.0002	0.805	− 0.002	0.002
Northern Territory	(0,0,1)	19.35	0.88	0.001	0.559	− 0.003	0.01
Queensland	(1,0,1)	29.12	0.40	− 0.003	0.519	− 0.01	0.01
South Australia	(0,0,1)	34.74	0.17	0.0003	0.734	− 0.001	0.002
Western Australia	**(0,0,0)**	**20.78**	**0.83**	**0.01**	**0.009**	**0.002**	**0.02**
Victoria	(1,0,0)	24.55	0.65	0.00002	0.99	− 0.003	0.003

### Homicide

**Fig. 8:**
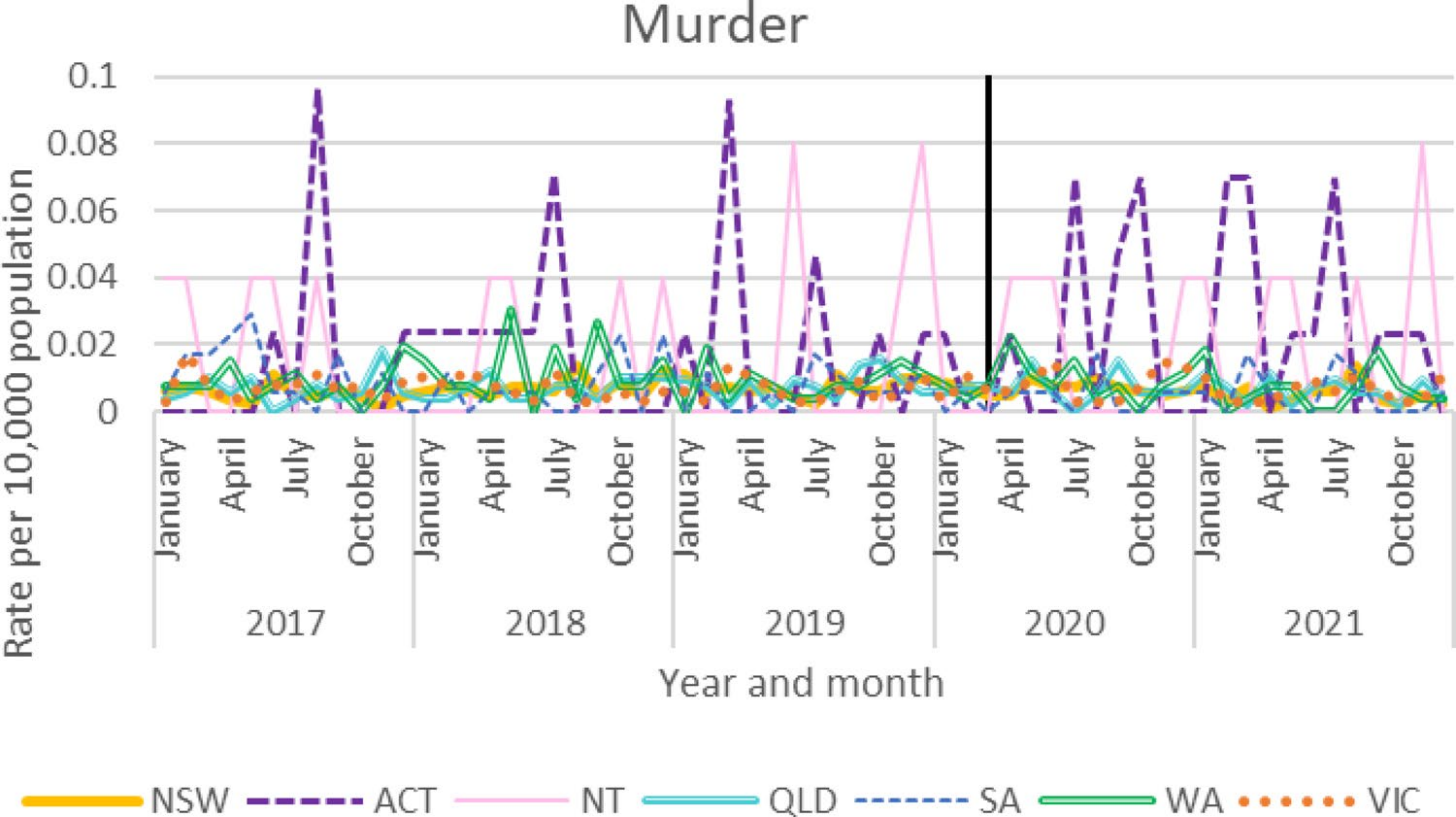
Homicide rates by state

**Fig. 9:**
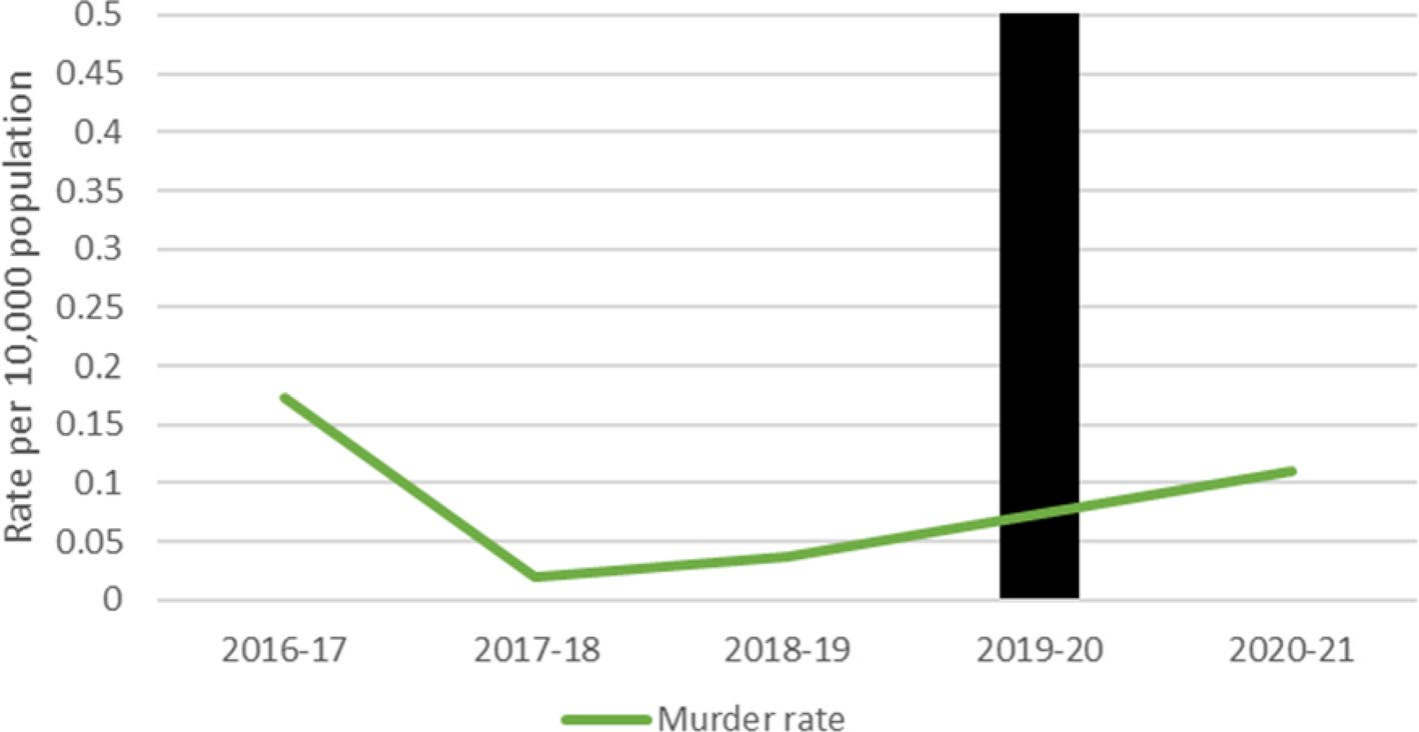
Homicide rates in Tasmania

Monthly homicide offence rates for all jurisdictions are presented in Fig. 8. As Tasmanian data reported on the basis of the financial year, this is presented in Fig. 9. Monthly homicide rates remained stable across the period examined. Due to floor effects, no analysis was conducted.

#### Tasmania Homicide Rates

Financial yearly homicide rates remained stable in Tasmania across the period examined.

## Discussion

The current paper investigated trends in violent offences pre- and post-COVID-19 to identify potential impacts of pandemic-related restrictions throughout Australia. Overall, violent offences remained relatively stable with brief fluctuations in lockdown periods. Specifically, most Australian jurisdictions have seen a continued gradual increase in police-recorded domestic assaults between 2017 and 2021, excluding the Northern Territory where there was an overall reduction pre-COVID-19. In the NT, there was no statistically significant impact of COVID-19 restrictions on domestic assaults, but there was a substantial increase after bio-security lockdowns were repealed in the second half of 2020 and people from ‘alcohol-free’ communities returned to larger centres where alcohol was available despite substantial restrictions [44]. Whilst family violence support services across Australia reported increased pressure during the pandemic, it may be that police-recorded assaults did not show the types of incidents driving calls to the support services.

Our findings align with a range of studies which found that whilst formally recorded domestic-violence-related offences and police involvement decreased, calls to domestic violence hotlines may have increased [14, 20], although we have no independent data to confirm this. On the other hand, our findings differ to initial studies in Europe, Asia, China and America which reported that family and domestic violence prevalence increased since the COVID-19 pandemic [11, 13–19]. Other research has reported that there was a spike in family and domestic violence calls immediately after a lockdown, before returning to relatively normal levels [18, 19, 21, 22] and one study from Japan found sexual assault victimisation significantly decreased during lockdowns [23]. These studies tend to show a wide range of trends in different countries, which may be related to several variables such as stringency of control measures implemented by governments, the amount of financial support given to individuals and families during this time, the levels of health care and law enforcement personnel able to assist people experience stress, and the effectiveness of already existing violence reduction measures such as integrated service network responses [45] and Violence Reduction Units [46]. For example, whilst the current Australian responses to family violence based on the Duluth model (such as Men’s Behaviour Change programmes which aim help men to examine their belief system and behaviours that support violence) have been found to be ineffective in reducing actual perpetration [47], integrated service network models such as Project Drive from the United Kingdom, which focuses on identifying and addressing the criminogenic needs of perpetrators and support needs of the family through a case-managed support network, has been found to reduce physical abuse by 82%, sexual abuse by 82% and jealous and controlling behaviours by 73% [45].

Non-domestic assaults in most Australian jurisdictions showed stable or slight downward trends over the full study period, except for the NT and WA. These two jurisdictions saw apparent trends increasing after the initial lockdown-related declines. A number of possible reasons might be suggested, and indeed, a combination of factors most likely explains many of the trend changes. Both jurisdictions are comparatively remote and implemented strict border controls, ‘locking out’ most visitors and allowing local residents to return to a mostly normal life whilst the virus spread rapidly through NSW and then Victoria. On the other hand, some people in the more remote areas may have experienced heightened frustration and isolation during lockdowns, or it may be that these communities experienced increased social pressures. Another possibility is that some of this harm was due to the increased financial access to alcohol in remote communities, which likely undermined existing price and availability control measures already in place [48]. Both Western Australia and Northern Territory also have a high proportion of remote communities, where many First Nations people experience the inter-generational trauma effects of colonisation and ongoing lack of access to adequate resources and supports. There were specific fears that if the COVID-19 virus was to enter remote communities, the impacts would be much worse than the general population. The NT, in particular, introduced substantial community isolation measures and returned many people to these communities from larger centres (voluntarily) as a health-protection measure.

The generally stable trends were consistent with the findings of Payne and colleagues’ paper which tracked violent crime in the pandemic, and found that none of the four violent crime categories was significantly different from the forecast, and, in fact, the rate of recorded serious assault and sexual offending was significantly lower in April [25]. However, the inclusion of data from states other than Queensland, which allows for the differentiation between domestic and non-domestic violent offences, does not support their hypothesis that a decrease in violence associated with the night-time economy may be offset by increases in assaults in residential settings, especially domestic violence. Rather, the longer trends show that when nightlife is closed, there are less non-domestic assaults, but this was not displaced to the home environment, potentially because many of the people in the relevant age group that attend nightlife do not have domestic partners at home. This fits somewhat with previous research which has highlighted that more than half the offences occurring on the street have been associated with licenced premises in Australia [49], although it is also likely that more general interactions in public spaces (e.g. public transport) contributed to reduced levels of violence as well. Alternatively, it may be that the availability of alcohol is relevant in these trends, because whilst nightlife was shut down and alcohol was not available in that context, it became far more available (with some limits) due to the explosion of home delivery services. Off-premise outlets were classified as ‘essential services’ in all jurisdictions, but some volume limits were mandated in WA because of hoarding fears. These findings are of interest when compared to those from South Africa, where a much stronger stance was taken on alcohol sales and a sudden and unexpected nation-wide alcohol sales ban reduced injury-induced mortality in the country by at least 14% during the 5 weeks of the ban [50]. They found more specifically that homicides, assault with intent to inflict grievous bodily harm and rape dropped during the alcohol ban period, followed by a large increase once alcohol was again available [50]. Unfortunately, their data were not able to distinguish between domestic and non-domestic violence.

### Limitations

Police data substantially underrepresent violent offences. Our previous work has identified that up to 60% of violent episodes are not reported to police [51]. However, police data is a reliable data source for assessing trends over time, presuming that there are no changes in the data collection/recording process. As documented, this occurred in Queensland in July 2021 where police practise changed in response to recommendations from a royal commission into family violence to ensure that assaults in the domestic context are now recorded as assaults in the general assault numbers, meaning that there was a substantial and sustained increase in recorded assaults reported. In the context of COVID-19, it may be that people were less likely to call police due to fear of transmission of the virus, but there is no way to be sure if this occurred. An alternative hypothesis here is that some violence may be about because of the need to physically or psychologically control the movements of partners/family members. If this is the case, then the lockdown period may have provided a context (because of restricted movements) where the need to use violence in this manner was not escalated. Additionally, the current paper examined medium-term trends. Nevertheless, it may be that the impacts of the pandemic on violence trends are more protracted and long term, although it remains helpful to document medium-term trends to inform public discussion. This is especially important when so many other policy changes (social, financial and law enforcement) are occurring which may impact on trends as well as potentially mask or alter community trends. Therefore, further research is needed to explore possible ongoing effects.

## Conclusion

There does not appear to have been a ‘dual pandemic’ as hypothesised in Australia. Whilst domestic violence rates have continued to rise, non-domestic violence rates have been trending down for the most-part, and the national lockdown implemented at the start of the pandemic saw a short-lived reduction in assaults outside the home. There are a number of potential explanations for the minor fluctuations observed, such as reduced interactions in public and the possibility that less violence was reported to police due to lack of opportunity. On the other hand, the lack of change in violence in the home may have also been partially related to increased alcohol availability increasing in the home due to home delivery services. Given the ongoing increasing and high levels of domestic violence in Australia, revised conceptual frameworks regarding the prime drivers of violence are indicated and interventions which address the criminogenic needs of offenders (e.g. mental health, trauma and substance use) warrant independent scientific evaluation [45].

## Data Availability

The datasets analysed during the current study are available, and were derived from, the following public domain sources. New South Wales: https://www.bocsar.nsw.gov.au/Pages/bocsar_datasets/Offence.aspx. South Australia: SAPOL—Crime statistics (police.sa.gov.au). Victoria: Download data | Crime Statistics Agency Victoria. Northern Territory: Northern Territory Crime Statistics January 2022—Datasets—NTG Open Data Portal. Western Australia Police Force Crime Statistics | Western Australia Police Force. Queensland: Maps and statistics | QPS (police.qld.gov.au). Australian Federal Police: https://www.policenews.act.gov.au/crime-statistics-and-data/crime-statistics.

## Abbreviations

ACT: Australian Capital Territory
ARIMA: Autoregressive Integrated Moving Average
GBH: Grievous bodily harm
NSW: New South Wales
NT: Northern Territory
COVID-19: SARS-CoV-2
SA: South Australia
WA: Western Australia
